# Prevalence and association of perceived stress, substance use and behavioral addictions: a cross-sectional study among university students in France, 2009–2011

**DOI:** 10.1186/1471-2458-13-724

**Published:** 2013-08-06

**Authors:** Marie Pierre Tavolacci, Joel Ladner, Sebastien Grigioni, Laure Richard, Herve Villet, Pierre Dechelotte

**Affiliations:** 1 Rouen University Hospital, Clinical Investigation Center CIC 0204, 1 Rue de Germont, Rouen Cedex 76031, France; 2 Inserm, U1073, Rouen University Hospital, Rouen, France; 3 Department of Epidemiology and Public Health, Rouen University Hospital, Rouen, France; 4 Department of Nutrition, Rouen University Hospital, Rouen, France; 5 Regional Observatory of Health, Rouen, France

## Abstract

**Background:**

University students face multiple stressors such as academic overload, constant pressure to succeed, competition with peers as well as concerns about the future. Stress should not be considered on its own, but should be associated with potential risk behaviors leading to onset of substance use and related problems heightened during the university period. The aim of this study was to determine the prevalence of main substance use and behavioral addictions among students in higher education in France and to examine the relationship with perceived stress.

**Methods:**

A self-administered questionnaire was filled out by university student volunteers from Upper Normandy (France) either by anonymous online questionnaire or by paper questionnaire. Data collected included socio-economic characteristics, Perceived Stress Scale (PSS), substance use (tobacco, alcohol, and cannabis) and hazardous behaviors: alcohol abuse problems, smoking, consumption of cannabis, eating disorders, and cyber addiction.

**Results:**

A total of 1876 students were included. Mean PSS score was 15.9 (standard deviation = 7.2). Highly stressed students (4th quartile) were compared with lesser stressed students (1st quartile). A positive relation was observed between female gender, regular smokers, alcohol abuse problems, risk of cyberaddiction and especially eating disorders (AOR = 5.45, 95% CI = 3.42-8.69), and increasing PSS score. PSS score however, was not significantly related to the curriculum, regular alcohol use, drunkenness or binge drinking even after additional controlling for use of other substances. We found a significant negative association between stress and practice of sport: students with the most physical activity were less likely to report perceived stress (4th quartile: AOR = 0.57, 95% CI = 0.39-0.80).

**Conclusions:**

This cross-sectional study among university students in France revealed that perceived stress was associated not only with known risks such as alcohol misuse, but also with new risks such as eating disorders and cyber addiction. These results could help to develop preventive interventions focussing on these risk behaviors and subsequently improving stress coping capacity in this high-risk population.

## Background

Stress is a term frequently used in a variety of social, academic and employment settings. Everyone needs a certain amount of pressure to perform at their best. However, when pressure exceeds a person’s ability to cope, this results in stress. Moreover, stress can set up a cycle of distress and reduce ability to cope even in ordinary situations. In today’s ultra competitive environment, students face more stress than ever, be it related to studying, examinations, or peer, teacher or parental pressure [1]. University students, often experience undue amounts of stress, which can have negative academic, emotional and health outcomes [2]. They face multiple stressors such as academic overload, constant pressure to succeed, competition with peers and in some countries financial burden as well as concerns about the future [3]. As all this may lead to psychopathology, the health of university students, especially healthcare students, has been the subject of increasing focus in recent years [4,5]. Voltmer *et al*. reported a decrease in quality of health and an increase in risk patterns, indicating a need for prevention and health promotion focussing on individual behavior [6].

In addition, common stressful life events are associated with both mental health symptoms and substance use in young adolescents [7]. Therefore, stress should not be considered on its own, but rather should be associated with potential risk behaviors. Moreover, the risk of onset of substance use and related problems is heightened during the university period. The most common substances used by young adults are alcohol, tobacco and cannabis [8-10]. The use of two or more of these substances simultaneously, known as polydrug use, has received growing attention in the literature due to an increase in prevalence in early adulthood [11]. Onset of smoking and alcohol drinking during early adulthood is a well-documented and significant public health risk factor, and is linked to a high risk of chronic diseases at older age [12-14]. Furthermore, substance use is associated with immediate health problems such as academic difficulties, injuries, interpersonal violence [15], high-risk sexual behavior [16], depression, and mental disorders [17]. Alcohol misuse in students has led to specific behavior known as binge drinking [18-20].

Stress in young adults is also often associated with the occurrence of substance-free addictions (also called behavioral addictions), such as cyberaddiction, addiction to sex, workaholism, and addiction to shopping [21-23]. Problematic internet use and addiction is characterized by excessive urges or behaviors regarding internet use, leading to impairment or distress among adolescents and young adults [24]. Risk behavior and associated stress mainly related to tobacco, alcohol and cannabis use [7,25], have already been widely reported in a population of healthcare students [1,2,26,27]. However, eating disorders and risk of cyber addiction have never been associated with stress in university students. In especially stressful situations, students often have an inaccurate perception of body weight, which can trigger abnormal eating behavior and eating disorders in predisposed individuals. Sassaroly *et al*. reported that isolated stressful situations might induce dieting, binging and compensatory behaviors in female students with no previous clinical eating disorders, but who were dissatisfied with their bodily aspect [28].

Hence, the purpose of this study was to determine the prevalence of main substance use and behavioral addictions among university students in France and to investigate the relationship between substance use, behavioral addictions, and stress in order to implement appropriate preventive interventions to foster public health benefits.

## Methods

### Setting

Our health educational program focused on students in higher education aged between 18 and 25 years and on seven campuses in Upper Normandy (France). A dynamic partnership was formed with the regional multidisciplinary group “Ta Santé en 1 Clic” (TS1C), involving ten partners from the health, higher education, and voluntary sectors as well as peer-students, all actively involved in the project design. The peer-students received two days of training by experts. The TS1C program was based on two main actions: Health Forums on campus led by peer-students and a website specifically dedicated to students: http://www.tasanteenunclic.org. Students were invited to participate in the survey during the health forum on campus. Student behavior prior to the health forums was assessed either by anonymous online questionnaire on the TS1C website or by paper questionnaire.

### Data collection

The self-questionnaire was anonymous and included socio-economic characteristics (age, gender, size, weight, grant holder status, student job status, lifestyle), regular practice of a sport, substance use and disorders (tobacco, alcohol, cannabis, and eating habits), and risk of cyberaddiction and perceived mental stress (Additional file 1).

The study was carried out between 2009 and 2011. The universities and schools involved were specialised in various areas: technologist, business, engineering; psychology, sciences, language and sport were regrouped in university; medicine and pharmacy; nursing, physiotherapy, midwifery and radiologic technologist studies were regrouped in para–medic. The questionnaire did not include any information that could be used to identify the students. Data collection was anonymous and confidential. Moreover, written informed consent was systematically obtained from students. The study design was approved by the “Commission Nationale de l’informatique et des libertés” (The French Data Protection Authority) (Additional file 2).

#### Stress measurement

*The Perceived Stress Scale* (*PSS)* was developed to measure the extent to which recent life status was appraised as stressful [29]. There are three versions of the PSS: with 4, 10, and 14 items respectively. The translation into French, which was validated by Koleck and Quintard corresponds to the 10-item version [30] and was used in this study. Each item was scored from 0 (*never*) to 4 (*very frequent*), and scores of items 4, 5, 7 and 8 were reversed. Higher scores corresponded to higher perceived stress (linear relation). The PSS is not a diagnostic instrument, so there were no cut-offs to determine stressed individuals and only comparisons between individuals were allowed. In our study, stress level was analysed by quartiles of perceived stress: The first quartile represented the less stressed students, with a PSS score of less than 10, the second quartile had a PSS score of 10 to 15, the third quartile had a PSS score of 16 to 20, and the fourth quartile represented highly stressed students with a PSS score of more than 20 to allow for nonlinear effects. The first quartile served as the reference group for analysis.

#### Assessment of substance use

The study focused mainly on the three most common psychoactive substances used by students: tobacco, alcohol and cannabis. In France, regular smokers are defined as smoking at least one cigarette per day, and regular alcohol and regular cannabis users are defined respectively as drinking alcohol at least 10 times per month, and using cannabis at least 10 times per month [31,32]. From these thresholds, polydrug user status in our sample was defined as follows: polydrug use referred to someone who, on a regular basis, uses at least two of the three most used psychoactive substances: tobacco, alcohol and cannabis.

According to the definition of the National Institute of Alcohol Abuse and Alcoholism (NIAAA), prevalence of binge drinking behavior was determined as five or more alcoholic drinks (four or more for women) on any one occasion and at least once a week [33]. In 2003, Naimi *et al*. defined regular drunkenness as a frequency of at least 10 times of acute drunkenness per year [33]. Substance use disorders, such as binge drinking, regular drunkenness were collected, and alcohol misuse were assessed by ADOSPA test [34]. ADOSPA (ADOSPA is the acronym in French of Auto, Détente, Oubli, Seul, Problème, Ami) is the French version of the CRAFFT (acronym of Car, Relax, Alone, Forget, Family/Friends, Trouble) questionnaire [35]. This questionnaire is a mnemonic screen tool to identify alcohol abuse problems in young adults and contains six items that describe the use of alcohol to relax, use of alcohol when alone, drinking alcohol and driving or riding with an alcohol drinking driver or rider, family or friends’ concern over alcohol use and, experiencing negative consequences of drinking. A score of two or more positive items usually indicates an alcoholic disorder.

##### Eating disorders and health

The SCOFF (Sick, Control, One stone, Fat, Food) questionnaire is a screening tool used to identify eating disorders, including anorexia nervosa and bulimia nervosa, in young adults. The SCOFF questionnaire includes five dichotomous questions, and one point is given for every “yes” answer. SCOFF is scored from 0 to 5 [36]. A score of 2 indicates possible anorexia nervosa or bulimia nervosa. Our team has recently validated the French version (SCOFF-F) of the SCOFF questionnaire and obtained an accurate and reliable tool for the detection of eating disorders in a French speaking student population [37].

#### Risk of cyberaddiction

Dr. Orman, author of the Internet Stress Scale, assessed the tendency of becoming a net addict in a 9-item test (Valleur *et al*. 2002) [38]. Students with more than four positive answers were classified as having a risk of cyberaddiction.

### Statistical analysis

The Chi-square (χ^2^) test or Fisher’s exact test were used for comparisons of qualitative data. Quantitative variables were summarized by means and medians and compared using the Student’s t-test. Logistic regression was performed to evaluate the independent determinants of perceived stress. Crude Odds Ratios (OR) and Adjusted Odds Ratios (AOR) and their 95% confidence intervals (CI) were calculated. Factors with a *p* value lower than 0.25 were included in the multivariate analysis and *p* value lower than 0.05 was considered to be significant. Statistical analysis was conducted using Statview® software package.

## Results

### Baseline characteristics of the study population

A total of 1876 students were included in the study between April 2009 and December 2011. The baseline characteristics and PSS score for each group are described in Table 1. The mean (Standard Deviation (SD)) age of students was 20.8 years (SD = 2.1) with a sex-ratio M:F of 0.51.

**Table 1:** Baseline characteristics of the 1876 students included and Perceived Stress Scale mean scores 2009-2011

	**Number**	**%**	**PSS score Mean (SD)**	**P**
**Age**				
18-19 years	514	27.4	16.3 (7.1)	0.34
20-21 years	800	42.6	15.8 (7.1)	
>21 years	562	30.0	15.6 (7.4)	
**Gender**				
Male	635	33.9	13.2 (6.5)	<10^-3^
Female	1239	66.1	17.3 (7.1)	
**Curriculum**				
Business	511	27.2	15.4 (7.0)	0.03
University	333	17.7	16.9 (7.7)	
Engineering	378	20.1	16.3 (7.1)	
Paramedic	217	11.5	15.8 (7.0)	
Medicine and pharmacy	148	7.8	15.4 (7.3)	
Technologist	214	11.4	15.0 (6.7)	
Others	77	4.1	16.0 (7.3)	
**Living in couples**				
Yes	258	14.3	16.5 (7.3)	0.39
No	1602	85.7	15.8 (7.2)	
**Student job**				
Yes	424	22.7	15.8 (7.3)	0.68
No	1446	77.3	16.0 (7.1)	
**Study grant holder**				
Yes	519	27.7	16.3 (7.3)	0.23
No	1352	72.3	15.8 (7.2)	
**Students living**				
At parents	530	28.5	15.6 (7.1)	0.42
In rented accomodation	1010	54.3	15.9 (7.3)	
On campus	321	17.2	16.5 (7.0)	
**Practice of sport**				
Yes	1154	62.1	15.2 (7.1)	<10^-3^
No	705	37.9	17.0 (7.7)	

Abbreviation: *SD* Standard deviation.

### Prevalence of substance use and disorders

Regular consumption of tobacco, alcohol and cannabis is described in Table 2. Tobacco was the most common substance used in our population, with 23.2% of students smoking at least one cigarette a day. This was followed by alcohol, with 20.1% of students drinking alcohol more than 10 times a month, and then cannabis with 3.7% of students consuming cannabis more than 10 times per month. Polydrug use concerned 8.9% of students. Regarding high risk alcohol consumption, 32.5% of the study population misused alcohol according to the ADOSPA questionnaire, with 18.7% of students experiencing frequent bouts of drunkenness. The prevalence of binge drinking (at least once a week) was 16.3%. Regarding BMI, 8.8% of students were overweight (BMI over 25), and 9.7% were underweight (BMI between 18 and 21). Dieting concerned 28.8% of students, most of whom were women (85.6%). SCOFF was positive for 22.2% of students (83.7% women). Prevalence of cyberaddiction risk was 26.9%.

**Table 2:** Frequencies of substance use, disorders and Perceived Stress Scale score in students (N = 1876) 2009-2011

	**Number**	**%**	**PSS score Mean (SD)**	**p**
**Regular smoker** (≥1cigarette per day)				
Yes	435	23.2	16.9 (7.2)	0.03
No	1436	76.0	15.7 (7.2)	
**Regular cannabis user**				
Yes	70	3.7	15.6 (6.9)	0.70
No	1808	96.3	15.9 (7.4)	
**Regular alcohol user**				
Yes	374	20.1	14.5 (6.7)	<10^-3^
No	1489	79.9	16.3 (6.9)	
**Polydrug user**				
Yes	167	8.9	15.8 (7.2)	0.82
No	1711	91.1	15.9 (7.3)	
**Alcohol abuse problems**				
**Positive ADOSPA**				
Yes	541	32.5	16.4 (7.1)	0.009
No	1179	68.5	15.4 (7.0)	
**Binge drinking**				
Yes	306	16.3	14.5 (6.7)	<10^-3^
No	1571	83.7	16.4 (7.1)	
**Drunkeness >10 per year**				
Yes	310	18.7	14.5 (6.7)	0.001
No	1348	81.3	16.0 (7.1)	
**Risk of eating disorders (positive Scoff)**				
Yes	417	23.2	20.2 (7.1)	<10^-3^
No	1428	76.8	14.7 (6.7)	
**Risk of cyber addiction (Orman test)**				
Yes	505	27.5	17.9 (7.2)	<10^-3^
No	1333	72.5	15.1 (7.0)	

Abbreviation: *SD* Standard deviation.

### PSS score with characteristics of the study population

Mean PSS score was 15.9 (SD = 7.2, median = 16.0, extremes = 0-40) in the 1876 students. Concerning baseline characteristics, PSS scores differed according to gender, curriculum and practice of sport: female university students were significantly more stressed (Table 1). Conversely, students practising sport were less stressed. There were no significant differences related to age, living and housing conditions, student grant holder status or student job status (Table 1).

Regular consumption of cannabis and polydrug use were not significantly associated with stress (Table 2). However, there were significant differences in alcohol and tobacco users: regular alcohol users had lower PSS scores whereas regular smokers had higher PSS scores. All risk behaviors were significantly associated with PSS scores.

Table 3 presents stress levels per quartile according to student characteristics as well as frequencies of substance use and disorders. Logistic regression analyses compared highly stressed students (4th quartile) with lesser stressed students (1st quartile): a positive relation was observed between female gender, regular smokers, ADOSPA test, risk of cyberaddiction and especially eating disorders (AOR = 5.45, 95% CI = 3.42-8.69) (Table 4), and increased PSS score. Conversely PSS score, was not significantly related to the curriculum, regular alcohol use, drunkenness or binge drinking even after additional controlling for use of other substances. Our results revealed a significant negative association between stress and practice of sport. Students with the most physical activity were less likely to report perceived stress (4th quartile: AOR = 0.57, 95% CI = 0.39-0.80).

**Table 3:** Risk factors associated with stress per quartile (N = 1876) 2009-2011

	**PSS score quartiles**				**P**_**trend**_
	**Q1 [0–09]**	**Q2 [10-15]**	**Q3 [16-20]**	**Q4 [>20]**	
**Sex Ratio**	1.10	0.67	0.45	0.19	<10^-3^
**Curriculum**					
Technologist %	21.4	34.4	26.8	17.3	0.02
Business %	22.8	31.9	25.2	29.3	
University %	18.2	21.4	31.8	28.6	
Engineering %	18.4	27.4	26.6	27.5	
Paramedic %	17.0	30.9	26.3	25.8	
Medicine and pharmacy %	26.9	24.6	21.5	26.9	
Other schools %	20.7	22.4	27.6	29.3	
**Study grant holder** %	26.2	27.4	24.9	30.9	0.25
**Practice of sport** %	72.6	63.3	63.3	44.2	<10^-3^
**Regular smoker (≥1cigarette per day)** %	20.8	21.3	29.3	27.6	0.007
**Regular alcohol user** %	26.5	24.8	19.1]	16.8	0.002
**Binge drinking** %	20.2	21.1	16.1	13.6	0.01
**Alcohol abuse problems (Positive ADOSPA test)** %	17.3	27.3	18.2	13.4	0.07
**Drunkenness >10 per year** %	21.6	22.5	25.8	17.5	0.01
**Risk of eating disorders (positive Scoff)** %	8.1	14.8	22.3	43.7	<10^-3^
**Risk of cyber addiction (Orman test)** %	17.8	24.51	28.6	36.2	<10^-3^

**Table 4:** Risk factors associated with perceived stress by quartile (logistic regression) (N = 1876)

	**Q1 [0–09]**	**Q2 [10-15] AOR (95% CI)**	**p**	**Q3 [16-20] AOR (95% CI)**	**p**	**Q4 [>20] AOR (95% CI)**	**p**
**Male**	1.00	0.58 [0.41-0.81]	0.0014	0.40 [0.28-0.57]	<10^-3^	0.18 [0.11-0.26]	<10^-3^
**Curriculum**							
Technologist (Ref)	1.00	1.00		1.00		1.00	
Business	1.00	0.77 [0.46-1.30]	0.34	0.69 [0.39-1.21]	0.19	0.78 [0.40-1.51]	0.46
University	1.00	0.62 [0.35-1.11]	0.11	1.15 [0.64-2.08]	0.63	1.41 [0.72-2.79]	0.31
Engineering	1.00	0.88 [0.52-1.52]	0.66	1.02 [0.58-1.80]	0.93	1.57 [0.82-3.02]	0.17
Paramedic	1.00	0.93 [0.49-1.77]	0.83	0.81[0.42-1.59]	0.06	1.11 [0.53-2.34]	0.78
Medicine and pharmacy	1.00	0.50 [0.26-1.00]	0.05	0.51 [0.25-1.04]	0.06	1.06 [0.49-2.32]	0.87
Other schools	1.00	0.71[0.28-1.80]	0.47	1.08 [0.43-2.74]	0.86	1.71 [0.61-4.81]	0.31
Study grant holder	1.00	1.04 [0.74-1.46]	0.82	0.85 [0.60-1.22]	0.40	1.35 [0.92-1.97]	0.12
**Practice of sport**	1.00	0.70 [0.51-0.97]	0.03	0.71 [0.51-1.00]	0.05	0.57 [0.39-0.80]	0.001
**Regular smoker (≥1cigarette per day)**	1.00	0.98 [0.67-1.43]	0.92	1.62 [1.11-2.36]	0.01	1.57 [1.04-2.37]	0.03
**Regular alcohol user**	1.00	0.87 [0.57-1.31]	0.49	0.64 [0.42-1.00]	0.05	0.63 [0.39-1.02]	0.06
**Binge drinking**	1.00	1.24 [0.76-2.05]	0.39	1.01 [0.60-1.71]	0.97	1.12 [0.62-2.03]	0.69
**Alcohol abuse problems (Positive ADOSPA test)**	1.00	1.32 [0.91-1.91]	0.14	1.65 [1.12-2.42]	0.01	2.22 [1.46-3.35]	0.0002
**Drunkenness >10 per year**	1.00	1.05 [0.65-1.68]	0.85	0.93 [0.57-1.54]	0.79	0.77 [0.43-1.36]	0.37
**Eating disorders (positive Scoff)**	1.00	1.61 [0.99-2.61]	0.05	2.72 [1.42-3.64]	0.0007	5.45 [3.42-8.69]	<10^-3^
**Risk of cyber addiction (Orman test)**	1.00	1.58 [1.09-2.30]	0.01	2.02 [1.39-2.95]	0.0003	2.85 [1.90-4.28]	<10^-3^

## Discussion

To the best of our knowledge, this is the first study conducted in a large population of university students, in France, with its main focus on the relationship between perceived stress, substance use and related disorders.

Perceived stress among university students is gender related. Female students are more exposed to stress associated with risk of cyber addiction, disorders related to alcohol use, eating disorders, and regular smoking than their male counterparts. Stress levels in our study were higher among female students than male students. Whilst some previous studies found similar results to ours [39], others reported that gender was not a risk factor for stress [40].

Our results also revealed a high prevalence of students with risk of eating disorder, since almost a quarter of our population had positive SCOFF score. Previously, Costarelli reported similar prevalence [41]. Eating disorders are the main behaviors associated with perceived stress and increased risk in each stress quartile.

Significant results were found for alcohol use and perceived stress. Whereas stress scores were positively associated with some alcohol disorders, they were not associated with excess alcohol use (drunkenness and binge drinking). It is important to highlight that the rate of students with alcohol use disorders (positive ADOSPA test) increased with level of perceived stress, even after adjustment. These results suggest that high stress levels lead students, not only to hazardous drinking patterns, such as dependence or getting high, but also to harmful behaviors towards alcohol, regardless of quantity or frequency of consumption.

Cigarette smoking in students is often described as social smoking and most students smoke for reasons other than stress relief, especially to facilitate social interactions, to avoid feeling alone, and behaving as a member of a group [42]. During stressful events, cigarettes not only help stress management, but also express non-verbally to others that the student is stressed [43]. Student tobacco consumption in response to stress can be associated with increased risk of nicotine dependence in later life.

Risk of cyber addiction was 27.5%, which might seem high but not all students become cyberaddicted. The prevalence of internet addiction disorder in an Italian student population was 5% and 1% respectively for moderate and serious users [44]. While studies have indicated that internet addicts are mostly young males with introverted personalities, the rate is currently rising in the female population [45]. Our study revealed a positive trend towards increased stress levels in students at risk of cyber addiction. Excessive behavior in internet addicts may serve as a stress coping strategy, albeit inadequate. The Internet may also be used as a forum for expanding social networks and consequently improving the chance of meaningful relationships, self-confidence, social abilities, and social support. Those using internet primarily for online chatting believed that it was psychologically beneficial. They also believed that frequent internet users were lonely and that the Internet could be addictive. “Chat” users, who are socially fearful, may be using the Internet as a form of low-risk social approach and an opportunity to rehearse social behavior and communication skills, which may help them improve interactions in face-to-face social environments [46]. Nevertheless, addicts may use the internet for extended periods, isolating themselves from other forms of social contact, and focussing almost entirely on the Internet rather than broader life events [47].

Physical activity is also considered to have beneficial effects on mental health and stress coping capacity. Indeed, exercise as a moderator of stress levels was previously investigated in [48] university student populations [49]. Moreover, these results suggested an appraisal of physical activities in order to reduce psychological distress among students.

Although curriculum was not a risk factor for stress in our study, previous studies have shown that medical students are highly stressed and have proposed coping strategies [50]. To our knowledge, three recent nationally-representative surveys have studied the prevalence of substance use among young adults in France and allow comparisons [33,51,52]. Our results were similar to those reported by Riou Franca *et al*., for tobacco and cannabis, and Melchior *et al.* for alcohol, binge drinking and polydrug use. Common definitions and criteria concerning substance use need to be validated and used internationally to allow comparisons.

There is an attitude among students of turning a blind eye towards existing stress, which is a serious problem and may be a harbinger of serious mental and psychosocial problems. Based on the findings from our research, it is important for future prevention and intervention efforts to consider social setting and heightened stress among students as potential risk factors for engaging in risk behaviors.

Baker *et al*. found evidence of moderate rates of Internet use in the healthcare sector among adult internet users, as well as moderate effects of the Internet on the knowledge of users. Internet is clearly an important tool with the potential to improve information dissemination. The Internet is a means of transmitting information on health. The rise in popularity and possibilities of the Internet has led to a revolution in the provision of health-related information and treatment. While the health sector has primarily employed the Internet as a psycho-educational portal, advances in interactive technology have increased the potential of the medium to deliver targeted health interventions and other behavior change programs [49,53]. An interactive website: MyStudentBody.com offers a brief, tailored intervention to help heavy-drinking college students reduce their alcohol use. Acquiring and improving knowledge on student populations is a crucial factor in the development of health promotion programs in order to meet students’ needs and to help them cope. Enhanced understanding of the personality profile of university students can be helpful in academic and career choices. Developing efficient coping strategies in students and improving academic environments could also contribute to preventing the potential deleterious consequences of stress [50].

Our study has certain limitations in as much as students were invited to participate in the study, and therefore the sample was not randomized. However, since several study disciplines were represented, the sample might be considered as representative of the student population. In addition, this cross-sectional study was based on self-reported information provided by students and collected throughout the year with very few answers returned during the stressful examination period. There is also some potential for reporting bias which may have occurred because of the respondents' interpretation of the question or desire to report their emotions in a certain way or simply due to inaccuracies in responses. Despite these limitations, we consider that our study provides important new information on the multiple health risks related to stress in students.

## Conclusion

French-Normandy higher education students, especially female students, represent a stressed population with unhealthy risk behavior regarding nutrition, alcohol and Internet. Our findings stress the need to develop investigations in student populations, for better understanding of these new phenomena and to assess interactions with other risk behaviors. Prospective design studies are requiredto identify substance use and disorder changes in stressful situations. As a result, we are currently conducting the ELSE (Etude Longitudinale sur la Santé des Etudiants) longitudinal study with a cohort of undergraduate students to investigate both their levels of stress and factors associated with stress. Furthermore, new research areas are necessaryto investigate the impact of these risk behaviors on students and on their future professional careers.

## Competing interests

The authors declare that they have no competing interests.

## Authors’ contributions

All authors participated in the study conception and design. LR and MPT participated in data acquisition and extraction. MPT and JL performed statistical analysis. MPT, JL and PD performed interpretation of data. MPT drafted the manuscript. All authors contributed to critical revision and have read and approved the final manuscript.

## Pre-publication history

The pre-publication history for this paper can be accessed here:

http://www.biomedcentral.com/1471-2458/13/724/prepub

## Supplementary Material

Additional file 1 Study about the student’s health. Click here for file

Additional file 2 Récépissé de déclaration à la CNIL. Click here for file
