# Supplementary material for: Prevalence and association of perceived stress, substance use and behavioral addictions: a cross-sectional study among university students in France, 2009–2011
Source: BMC Public Health. 2013 Aug 6;13:724. doi: 10.1186/1471-2458-13-724 (PMC3750571; doi:10.1186/1471-2458-13-724)
Supplement: Additional file 1 — Study about the student’s health. [file 1471-2458-13-724-S1.doc]

**Study about the student’s health.**

This questionnaire is **anonymous**, the data are **confidential**.

All information received by the questionnaire is confidentially treated. Except the researchers of the study, who treat the information in the greatest respect for the confidentiality, the anonymity is totally preserved. The results of this study will used to scientific purposes and their publication doesn’t contain any individual result.

**YOUR SITUATION**

**How old are you?**__________ years

**What is your sex?**  Male   Female

**How tall are you?** *___________cm*

**What is your weight?** *___________kg*

**What is your nationality?**  French  Other

**What is your marital status?**  Single  Steady relationship (married, married life style…)

**Socioeconomic class of your father:**

 Farmer   Traders artisans

 Intermediate professionals (teacher, technician, nurse…)

 Employees    Worker  No occupation

 Executive and higher intellectual professions  Retired

**Socioeconomic class of your mother:**

 Farmer   Traders artisans

 Intermediate professionals (teacher, technician, nurse…)

 Employees    Worker  No occupation

 Executive and higher intellectual professions  Retired

**Your establishment/School: _________________________________________________**

**Studies followed:**  **Year:**

**Do you have a job during yours studies?**   YES  NO

**Have you a scholarship?**  YES  NO

**What kind of accommodation do you live in?**

 Rental/ Roommate  With my parents  university residence

**TOBACCO**

**You are:**  Smoker,

 Former smoker. If yes, how long? _________years

 Non-smoker

**If you are smoker:**

How many years have you smoked for? ___________

How many cigarettes do you smoke daily? ________

Do you intend to stop smoking?  YES  NO

If yes, what are your reasons to stop smoking?

 To preserve my health  Cigarettes are so expensive

 My family (friends) wants I stop it

 Other (*point out*): __________________________________________________

If you don’t want stop it, why?

 Not want to stop  Fear of becoming fat  Other (point out): ________________

Are you in an approach to smoking cessation?  YES  NO

If yes, with help (of someone and/or of products)  YES  NO

**CANNABIS**

**Have you ever consumed marijuana?**  YES  NO

If yes, how many times during:

The last 12 month: ________

The last 30 days: _______

**ALCOOL**

**How often do you consume drink containing alcohol?**

 Never  monthly or less  2 to 4 times a month  2 to 3 times a week  4 or more times a week

**How many drinks containing alcohol do you have on a typical day when you are drinking?**

 1 or 2  3 or 4  5 or 6  7 or 8 10 or more

**How often do you have six or more drinks (Beer 25cl, whisky 2,5cl…) on one occasion?**

 Never  Less than monthly  Monthly  Weekly  Daily or almost daily

**During your life, have you ever been drunk?**

 Never  Once or twice  3 to 9 times  10 or more times

If you have ever been drunk, how old were you the first time? ________years

**During the last 12 month, how many times have you been drunk?**

 Never  Once or twice  3 to 9 times  10 or more times

**Have you ever been in a vehicle (car, bike, scooter…) driven by someone (you including) who had drunk?**

 Never  Sometimes (< Monthly)  Often (> Monthly)

**Do you use alcohol to relaxed, to feel better or to hold out?**

 Never  Sometimes (< Monthly)  Often (> Monthly)

**How often do you drink alcohol when you are alone?**

 Never  Sometimes (< Monthly)  Often (> Monthly)

**Have you ever forgotten things what you had to do because of alcohol?**

 Never  Sometimes (< Monthly)  Often (> Monthly)

**Your family or your friends have you ever suggested to reduce your alcohol consumption?**

 Never  Sometimes (< Monthly)  Often (> Monthly)

**Have you ever had problems when you used alcohol?**

 Never  Sometimes (< Monthly)  Often (> Monthly)

**ALIMENTATION**

**How many fruits and vegetables do you consume daily?**

 0  1   2   3   4   5  >5

**How many servings of vegetables do you consume daily?**

 0  1   2   3   >3

How many times per week do you eat fats (French fries, kebab, donuts…)?

 0  1   2   3   4   5  >5

**Have you ever done a diet?**  YES  NO

If yes, how many times?  1  2 or 3   >3

| Do you make yourself sick because you feel uncomfortably full? |  OUI |  NON |
| --- | --- | --- |
| Do you worry you have lost control over how much you eat? |  OUI |  NON |
| Have you recently lost more than one stone in a 3 month period? |  OUI |  NON |
| Do you believe yourself to be fat when others say you are too thin? |  OUI |  NON |
| Would you say that food dominates your life? |  OUI |  NON |

**PHYSICAL ACTIVITY**

**Do you practice sport?**  YES  NO

If yes, what sport do you practice the most frequently? _______________________

How long per week? ___________hours

How many month per year ? ____________

**Compared with people of your age, you think your time dedicated to a physical activity is:**

 Much more  More  Similar  Less  Much less

How long, in a week (Monday, Tuesday, Wednesday, Thursday and Friday), during your recreation, you watch TV and/or internet? __________hours

How long, in a week-end (Saturday, Sunday), during your recreation, you watch TV and/or internet? ___________ hours

How long per day do you walk (except the practice of a sport)? _________ minutes

**STRESS**

# **Do you take anxiolytic drugs (against anxiety)?**  YES  NO

If yes:

How many?

1 to 7 times a week  1 to 4 times a month  less than 12 times a year

Do you take it before an exam (test, evaluation)?  YES  NO

# Do these drugs are prescribed by a doctor?  YES  NO

# **Do you take antidepressant?**  YES  NO

# Do these drugs are prescribed by a doctor?  YES  NO

**During the last month:**

**Have you been disturbed because of an unexpected event?**

 Never  Almost never  Sometimes  Often  Very often

**Has it seemed difficult to you to control the important things of your life?**

 Never  Almost never  Sometimes  Often  Very often

**Have you felt nervous or stressed?**

 Never  Almost never  Sometimes  Often  Very often

**Have you felt confident to take over your personal problems?**

 Never  Almost never  Sometimes  Often  Very often

**Have you felt that the things were going like you wanted?**

 Never  Almost never  Sometimes  Often  Very often

**Have you thought that you couldn’t assume all the things that you had to do?**

 Never  Almost never  Sometimes  Often  Very often

**Have you been able to control your nervousness?**

 Never  Almost never  Sometimes  Often  Very often

**Have you felt you dominated the situation?**

 Never  Almost never  Sometimes  Often  Very often

**Have you felt irritated because of the events were beyond your control?**

 Never  Almost never  Sometimes  Often  Very often

**Have you found that the difficulties accumulated to such an extent that you couldn’t control them.**

 Never  Almost never  Sometimes  Often  Very often

**INTERNET USE**

| Do you spend more time connected on internet that you would have thought? |  YES |  NO |
| --- | --- | --- |
| Are you annoyed by a limitation of your time on internet? |  YES |  NO |
| Have friends or family’s members complained about your time on internet? |  YES |  NO |
| Do you find difficult to not be connected on internet during few days? |  YES |  NO |
| Did your personal relationships and your studies suffer because of your time on internet? |  YES |  NO |
| Do you have particular websites that you can’t avoid on internet? |  YES |  NO |
| Do you have difficulties to control your impulse buying on internet? |  YES |  NO |
| Did you try, unsuccessfully, to reduce your internet use? |  YES |  NO |
| Do you think you have neglected sources of personal satisfaction because of internet? |  YES |  NO |

**SLEEP**

**During the last month:**

What time did you usually go to sleep the evening?

/____/ /_____/ h /____/ /____/

How many times did you need to fall asleep (in minutes)?

/____/ /_____/ minutes

What time did you usually wake up the morning? /____/ /_____/ h /____/ /____/

**During the last month, how often did you have sleep disorders because of:**

You couldn’t fall asleep in less than 30 minutes?

 Not in the last month  Less than once a week

 Once or twice a week  3 or 4 times a week  Everyday

You woke up in the middle of the night or precociously in the morning?

 Not in the last month  Less than once a week

 Once or twice a week  3 or 4 times a week  Everyday

During the last, how do you generally evaluate the quality of sleep?

 Very good  Good  Bad  Very bad

**Do you consume sleeping pills?**  YES  NO

If yes, how often:

 Not in the last month  Less than once a week

 Once or twice a week  3 or 4 times a week  Everyday

If yes, are they prescribed by a doctor?  YES  NO

**SOCIAL SITUATION**

| Do you sometimes meet a social worker? |  YES |  NO |
| --- | --- | --- |
| Do you have an additional health insurance? |  YES |  NO |
| Do you live in couple? |  YES |  NO |
| Are you homeowner? |  YES |  NO |
| Do you have period in the month where you meet real financial difficulties to meet your needs? |  YES |  NO |
| Have you ever practiced sport during the last 12 month? |  YES |  NO |
| Have you gone to a show during the last 12 month? |  YES |  NO |
| Did you go on vacation during the last 12 month? |  YES |  NO |
| During the last 6 month, did you have contacts with members of your family, except your parents? |  YES |  NO |
| In case of difficulties, do you have in your environment people who can count on to:  Host you few days if necessary?  Bring you a material aid ? |  YES   YES |  NO   NO |

**Thanks to take time to complete this questionnaire and to participate to this research.**
