# Supplementary material for: Prevalence and association of perceived stress, substance use and behavioral addictions: a cross-sectional study among university students in France, 2009–2011
Source: BMC Public Health. 2013 Aug 6;13:724. doi: 10.1186/1471-2458-13-724 (PMC3750571; doi:10.1186/1471-2458-13-724)
Supplement: Additional file 2 — Récépissé de déclaration à la CNIL. [file 1471-2458-13-724-S2.pdf]

Récépissé de déclaration

Numéro de déclaration
1353247

Monsieur Joel LADNER
CHU HOPITAUX DE ROUEN
DEPARTEMENT D'EPIDEMIOLOGIE ET DE SANTE PUBLIQUE
RUE GERMONT
DESP - HCN - CHU
76031 ROUEN

Conformément à la loi du 6 janvier 1978 relative à l'informatique, aux fichiers et aux libertés, modifiée en août 2004,

CHU HOPITAUX DE ROUEN
RUE GERMONT
DESP - HCN - CHU
76031 ROUEN

A déclaré à la Commission Nationale de l'Informatique et des Libertés un traitement automatisé d'informations nominatives dont la finalité principale est :

EVALUATION D'UNE INTERVENTION DE SANTE PUBLIQUE

La délivrance du présent récépissé ne vaut pas constatation de la conformité du traitement à la loi et n'exonère le déclarant d'aucune de ses responsabilités.

Paris, le 27 mars 2009
Par délégation de la commission

Alex Türk

Alex TÜRK
Président de la commission
